# Is It I?

**Published:** 1867-11

**Authors:** 


Is It I? A Book for Every Man. A Companion to Why Not? By Horatio Robinson Storer, M.D., of Boston, Vice-President of the American Medical Association. Boston: Lee & Shepard. 1867.

This is a duodecimo volume of 154 pages. The subjects treated of are:

1. It is not good to be alone.
2. Marriage as a Sanitary Measure.
3. How early in life is marriage to be advised?
4. The Rights of the Husband.
5. Are these Rights Absolute, or Reciprocal with Duties?
6. Should mere Instinct, or Reason, be the rule?
7. Arguments and Counter-Arguments as to Divorce.
8. A Plea for Woman.

These important topics are discussed by the author, in such a manner as to interest and profit the reader. Without sanctioning a certain vein of exaggeration, or *sensationalism*, which is perceptible in the author's style, we earnestly commend the work for general perusal. Its main object is to inculcate more correct views in relation to the effects of sexual intercourse, and to point out more clearly the proper relations between husband and wife, with a view to restraining the excesses of the former. Aside from the use of intoxicating drinks, there are probably no evils productive of more social misery than the abuse of the sexual propensities, both in the married and unmarried. Dr. Storer's little work can be found in all our bookstores.

